# Liberal versus restrictive transfusion strategies in patients with acute brain injury: a systematic review and meta-analysis of randomized controlled trials

**DOI:** 10.1080/08998280.2025.2586988

**Published:** 2025-12-10

**Authors:** AlMothana M. Manasrah, Mazen Alayidh, Ahmed A. Ibrahim, Ahmed A. Maiz, Mohamed Rifai, Shaden Alayidh, Sara A. Al Asheikh, Ali Alaklah, Mohamed Saad Rakab, Mustafa Turkmani, Mohamed Abuelazm

**Affiliations:** a Internal Medicine Department, UHS-Wilson Medical Center, Binghamton, New York, USA; b College of Medicine, King Khalid University, Abha, Saudi Arabia; c Faculty of Medicine, Menoufia University, Menoufia, Egypt; d College of Medicine, Alfaisal University, Riyadh, Saudi Arabia; e Eastern Health Cluster, Dammam, Saudi Arabia; f Faculty of Medicine, Mansoura University, Mansoura, Egypt; g Department of Pulmonary and Critical Care, University of Toledo, Toledo, Ohio, USA; h Faculty of Medicine, Michigan State University, East Lansing, Michigan, USA; i Faculty of Medicine, Tanta University, Tanta, Egypt

**Keywords:** Bleeding, emergency, hematoma, hemorrhage, packed red blood cells

## Abstract

**Background:**

Anemia has been observed in up to 46% of individuals with acute brain injury. Blood transfusions are commonly performed to raise hemoglobin levels, so we aimed to compare the restrictive and liberal blood transfusion strategies in acute brain injury patients.

**Methods:**

A systematic search was conducted on Web of Science, Embase, Scopus, Cochrane, and Medline/PubMed up to February 10, 2025. Continuous data were combined using mean differences (MD), and dichotomous outcomes were synthesized using risk ratios (RR); both were detailed with a 95% confidence interval (CI), applying R software (version 4.3). This study was registered and published with PROSPERO (ID CRD42025630392).

**Results:**

The analysis incorporated six randomized controlled trials involving 2599 participants. There were no substantial variations between the liberal and restrictive transfusion groups in unfavorable neurological outcomes (RR: 0.90 [95% CI: 0.79–1.03]; *P* = 0.13), favorable neurological outcomes (RR: 1.16 [95% CI: 1.00–1.35]; *P* = 0.05), hospital length of stay (MD: −0.66 [95% CI: −2.48–1.16]; *P* = 0.48), or intensive care unit length of stay (MD: 0.15 [95% CI: −0.68–0.99]; *P* = 0.72). However, the liberal transfusion strategy was accompanied with an increased number of red cell units transfused (MD: 2.28, 95% CI: [1.75–2.80]; *P* < 0.01) and reduced sepsis or septic shock compared to the restrictive strategy (RR: 0.73 [95% CI: 0.56–0.96]; *P* = 0.02).

**Conclusion:**

The liberal strategy of blood transfusion for patients with acute brain injury and anemia did not impact neurological outcomes. It reduced the incidence of sepsis or septic shock, but this came with an increase in the number of red blood cells transfused without affecting overall mortality or thrombotic events.

Traumatic brain injury (TBI) remains the leading cause of lasting disability and mortality worldwide. It affects an estimated 69 million individuals each year. Most cases are mild to moderate in severity.^1^ TBI results from various mechanical forces, including falls and fights, which typically cause transient mild to moderate symptoms. However, severe head trauma, often from road traffic collisions, can lead to long-lasting cognitive deficits, motor impairment, sensory loss, or emotional distress.^2^

Among individuals with TBI, anemia has been observed in up to 46% of patients.^3^ Studies have indicated that anemia is not the primary issue; when TBI patients have hemoglobin levels <9 g/dL, brain tissue oxygenation will likely decrease. This combination of anemia and compromised brain tissue oxygenation can lead to poorer outcomes within the first 30 days after severe TBI.^4^^,^^5^ Blood transfusions are commonly performed to raise hemoglobin levels, thereby improving cerebral tissue oxygenation and accelerating the recovery of brain function.^6^ However, transfusions carry risks, including infection, thromboembolism, respiratory failure, and death, especially in critically ill patients.^7^ There are two principal transfusion strategies: the liberal strategy, where transfusion is indicated if hemoglobin falls below 9 g/dL, and the restrictive strategy, where blood is given only when hemoglobin drops below 7 g/dL.^8^

Multiple studies have been conducted to determine whether the liberal or restrictive strategy yields better long-term outcomes. In 2006, McIntyre et al found no improvement in death rates among severely ill head trauma patients using the liberal transfusion approach compared to the restrictive one.^9^ In 2014, Robertson et al revealed that keeping hemoglobin levels above 10 g/dL did not enhance neurological endpoints over 6 months.^10^ In 2019, Gobatto et al found more favorable outcomes in the liberal group compared to the restrictive group.^5^ In 2024, Taccone et al supported Gobatto’s results, finding better neurological endpoints at 6 months with the liberal strategy.^8^ Nevertheless, Turgeon et al denied the superiority of the liberal strategy.^11^ Finally, the most recent randomized controlled trial (RCT), the SAHaRA trial, also couldn’t detect differences between the two strategies in neurological outcomes.^12^

To explore these conflicting results, this systematic review and meta-analysis was conducted to assess the impact on neurological outcomes of the restrictive and liberal blood transfusion methods to direct red blood cell (RBC) transfusions in individuals with TBI.

## METHODS

Our review was registered and published in PROSPERO under the ID CRD42025630392. We conducted a systematic review and meta-analysis guided by the Preferred Reporting Items for Systematic Reviews and Meta-Analyses (PRISMA) statement^13^ and the Cochrane Handbook of Systematic Reviews and Meta-Analysis.^14^ A PRISMA checklist is included in the *Supplemental Material*.

### Retrieval strategy and study selection

We searched Web of Science, PubMed (MEDLINE), Embase, Scopus, and the Cochrane Central Register of Controlled Trials (CENTRAL) databases up to February 10, 2025, employing customized keywords and MESH terms in each database. No search filters were applied. This timeframe was selected to ensure a comprehensive capture of all available RCTs on restrictive versus liberal transfusion thresholds in TBI without imposing arbitrary temporal limits. The comprehensive search methodology of each database is illustrated in *Supplemental Table S1.*

We incorporated research articles that adhered to the following PICO framework: P, Population, adults with acute brain injury as TBI, subarachnoid hemorrhage, or mixed cohorts, managed using transfusion; I, Intervention: liberal strategy (transfusion triggered by hemoglobin <9 g/dL or <10 g/dL); C, Control, restrictive strategy (transfusion triggered by hemoglobin <7 g/dL); O, Outcomes, favorable and unfavorable neurological endpoints evaluated by the extended Glasgow Outcome Scale (GOS-E), the Glasgow Outcome Scale (GOS), and the modified Rankin Scale (mRS), along with secondary outcomes of hospital and intensive care unit (ICU) length of stay, red-cell units transfused, mortality, sepsis or septic shock, pneumonia, hypotension, deep venous thrombosis, acute respiratory distress syndrome, and transfusion reaction.

The exclusion criteria for articles were as follows: (1) publications with overlapping or repeated datasets; (2) book chapters, letters to the editor, comments, reviews, and guidelines; (3) animal and in vitro studies; (4) observational studies to minimize bias and confounding; (5) studies involving pediatric populations, as transfusion practices and physiological responses differ from adults; (6) articles not written in English, due to limited resources for accurate translation; and (7) studies available only as grey literature, such as conference abstracts or theses, because of incomplete reporting and absence of peer review.

The web-based Covidence software was utilized to perform this systematic review. Following removal of duplicates from the search results, four authors (A.M.M, M.R., M.A., S.A.) separately evaluated each article. During the preliminary full-text screening to check the suitability and relevance of the included studies, the full texts of the articles were assessed by the same authors. Conflicts were settled by deliberation and consensus with another author (A.A.).

### Data extraction

Four authors (A.M.M., M.R., M.A., and S.A.) independently gathered data and entered them into a standardized Excel extraction form. Before commencing full extraction, two reviewers piloted the process using two randomly selected studies to ensure consistent interpretation of all variables, outcome definitions, and coding rules. The retrieved data included characteristics of the selected articles (country, publication year, last author name, number of centers, study design, main inclusion criteria, total number of patients, intervention details, control details, primary endpoint, and follow-up period), baseline patient characteristics (number of patients, gender, mean age in years, mean RBC units transfused before randomization, Acute Physiology and Chronic Health Evaluation II [APACHE II] score, subarachnoid hemorrhage, epidural hematoma, mean GOS, mechanism of injury, and admission laboratory findings), and efficacy and risk profile endpoints as stated previously. Any discrepancies were resolved through discussion among the primary extractors, with unresolved disagreements adjudicated by a senior author.

### Risk of bias and certainty of evidence

Three reviewers (A.M.A, E.H.M., Y.F.A) separately evaluated the quality of the included studies by utilizing the Cochrane RoB2 tool.^15^ They assessed five distinct domains: measuring the outcome, reporting selection, deviating from the planned intervention, missing results, and the random allocation bias. Any conflicts were addressed with a senior author. We employed the Grading of Recommendations Assessment, Development, and Evaluation (GRADE)^16^^,^^17^ process to determine the certainty of the evidence. The assessment was conducted for each endpoint, and the decisions were recorded and justified accurately. Any inconsistencies were resolved through discussion.

### Statistical analysis

Statistical analyses were performed using R version 4.3, applying the metafor, meta, and dmetar packages for statistical analysis. We assumed a high heterogeneity level and adopted a random-effects model, and forest plots were generated for each outcome. Furthermore, continuous data were analyzed as mean differences (MDs) with 95% confidence intervals (CIs), and dichotomous variables as risk ratios (RRs) with 95% CIs. *P* values <0.05 were taken as the level of significance. In cases where zero events were observed in both comparison groups for dichotomous outcomes, a continuity correction of 0.5 was applied. We carried out a random-effects model when marked heterogeneity (I^2^ > 50%) was identified utilizing the chi-square and I-square tests; otherwise, a common-effect model was applied, as outlined in the Cochrane Handbook.^14^ An I^2^ value of 0 to 40% suggests low heterogeneity, 30% to 60%, moderate heterogeneity; 50% to 90%, considerable heterogeneity; and 75% to 100%, substantial heterogeneity. A chi-square test *P* value < 0.1 signified statistical heterogeneity. Subgroup analysis was conducted by neurological outcome scale (unfavorable = GOS-E 1–4/1–5; GOS 1–3; mRS ≥4; favorable = complementary categories), and sensitivity analyses, including leave-one-out, were performed to detect the source of heterogeneity, particularly for transfusion units.

## RESULTS

**Figure 1.**
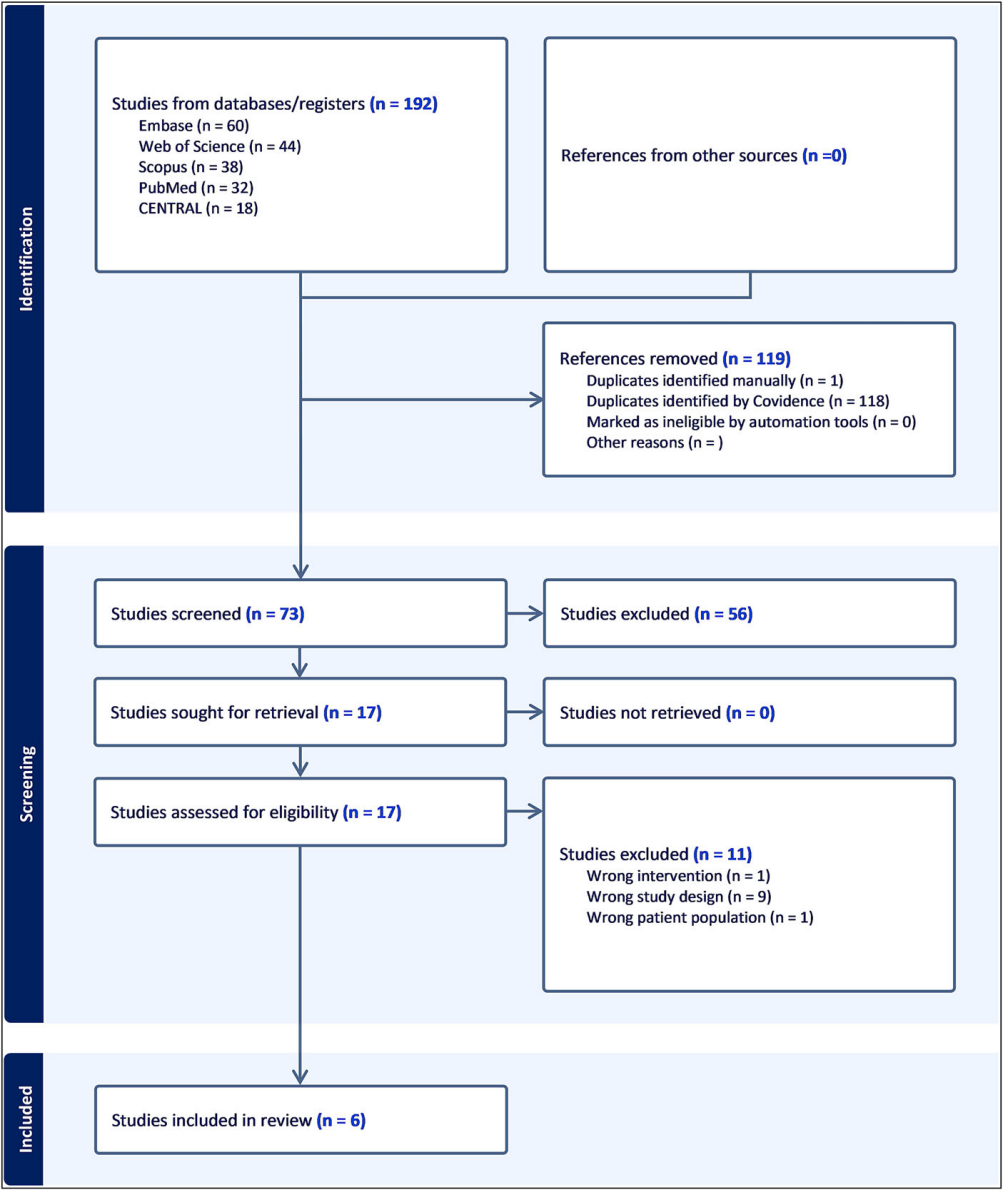
PRISMA flow chart of the screening process.

The combined database search revealed 192 articles from Scopus, PubMed, Web of Science, Embase, and Cochrane CENTRAL. After 119 duplicates were excluded by Covidence Online, 73 studies were screened. Of this group, 56 articles were removed by title and abstract screening. A total of 17 studies were shortlisted for eligibility, and full-text screening resulted in six eligible studies for our meta-analysis. *Figure 1* shows a PRISMA flow diagram of the study selection methodology and the database search approach.

### Characteristics of included studies

Six RCTs involving 2599 individuals were included in the statistical analysis, with 1292 patients in the liberal transfusion group and 1307 in the restrictive transfusion group. The sample sizes of the RCTs ranged from 44 to 820 patients. Liberal transfusion was defined as transfusion at a hemoglobin threshold of <9 or <10 g/dL, while restrictive transfusion was defined as transfusion at a hemoglobin threshold of ≤8 g/dL. The follow-up period was 6 months postrandomization in four studies; one study had a 2-month follow-up and another had a 12-month follow-up. An overview of the included RCTs is provided in *Table 1*, and the baseline characteristics of their participants are displayed in *Table 2*.

**Table 1.** Summary characteristics of the included randomized controlled trials

Study ID	Study design	Blinding	Country	Total participants	Intervention details		Main inclusion criteria	Follow-up duration	Primary outcome
					Liberal strategy	Restrictive strategy			
Gobatto et al^5^ (TRAHT)	Open-label, parallel, feasibility, RCT	Applied only to outcome assessors, not to ICU staff	Brazil	44	Hemoglobin transfusion threshold of 9 g/dL	Hemoglobin transfusion threshold of 7 g/dL	Patients >18 years admitted to ICU with moderate or severe TBI (GCS ≤12) and Hb <9 g/dL within 7 days from hospital admission	Up to 6 months after hospital discharge	Mean difference in Hb concentration between the groups during the 14 days postadmission
McIntyre et al^9^ 2006	Subgroup analysis of a multicenter RCT	Physicians were blinded to outcomes; patients were not blinded to Hb levels	Canada	67	Hemoglobin maintained at 10.0–12.0 g/dL; transfusions below 10.0 g/dL	Hemoglobin maintained at 7.0–9.0 g/dL; transfusions below 7.0 g/dL	Moderate to severe closed head injury, trauma, Hb <9.0 g/dL within 72 h of ICU admission	30 and 60 days after randomization	30-day all-cause mortality
Robertson et al^10^	RCT using a factorial (2 × 2) design	Investigators and clinicians blinded to assignment but not transfusion threshold; personnel conducting outcome assessments blinded to both	US	200	Transfusion of leukoreduced-packed RBC to maintain Hb ≥10 g/dL	Transfusion of leukoreduced-packed RBCs to maintain Hb ≥7 g/dL	Patients with closed head injury unable to follow commands after resuscitation, enrolled within 6 h of injury	6 months post-injury	Favorable vs. unfavorable neurological outcomes measured by GOS-E
Taccone et al^8^ (TRAIN)	Multicenter, phase 3, parallel-group, pragmatic, open-label RCT	Outcome assessor-blinded; participants and family members were blinded to the treatment assignment	22 countries	820	Transfusion triggered by Hb <9 g/dL	Transfusion triggered by Hb <7 g/dL	Adult patients (≥18 years) admitted to the ICU with (a) TBI, aneurysmal SAH, or ICH; (b) Hb <9 g/dL within 10 days of injury; (c) expected ICU stay ≥72 h	180 days	Proportion of patients with unfavorable neurological outcome (GOS-E score 1–5) 180 days post–randomization
Turgeon et al^11^ (HEMOTION)	Multicenter, RCT with a PROBE design	Endpoints were assessed in a blinded manner, though the trial itself was open label	Canada, the United Kingdom, France, and Brazil	736	Transfusions initiated at a Hb level of ≤10 g/dL	Transfusions initiated at a Hb level of ≤7 g/dL	Adults (≥18 years old) with moderate-to-severe TBI (GCS 3–12) and anemia (Hb ≤10 g/dL); patients screened during ICU admission	6 months post- randomization	Unfavorable neurological outcome assessed using the GOS-E, with a sliding dichotomy based on each patient’s baseline prognosis
English et al^12^ (SAHaRA)	Pragmatic, open-label, RCT	Outcome assessment was blinded	Canada, Australia, and the US	732	Mandatory transfusion at a Hb level ≤10 g/dL	Optional transfusion at a Hb level ≤8 g/dL	Adults (≥18 years) with first-ever aneurysmal SAH; Hb ≤10 g/dL within the first 10 days after admission	12 months	An unfavorable neurological outcome, defined as a mRS ≥4 at 12 mo

GCS indicates Glasgow Coma Scale; GOS-E, Glasgow Outcome Scale–Extended; Hb, hemoglobin; ICH, intracerebral hemorrhage; ICU, intensive care unit; mRS, modified Rankin Scale score; PROBE, Prospective Randomized Open Blinded End-Point; RBCs, red blood cells; RCT, randomized controlled trial; SAH, subarachnoid hemorrhage; TBI, traumatic brain injury.

**Table 2.** Baseline characteristics of the participants

Study ID	Number of patients		Age (years), Mean ± SD		Gender (Male), N (%)		Subarachnoid hemorrhage, N (%)		Epidural hematoma, N (%)		RBC units/patient before randomization, Mean ± SD		APACHE II score, Mean ± SD		Glasgow Coma Score, Mean ± SD		Mechanism of injury, N (%)								Laboratory findings on hospital admission (Mean ± SD)					
	Lib	Res	Lib	Res	Lib	Res	Lib	Res	Lib	Res	Lib	Res	Lib	Res	Lib	Res	Assault		Fall or jump		Motorcycle crash		Other		Glucose, mg/dL		Sodium, mmol/L		Hemoglobin, g/dL	
																	Lib	Res	Lib	Res	Lib	Res	Lib	Res	Lib	Res	Lib	Res	Lib	Res
Gobatto et al^5^ (TRAHT)	21	23	33 ± 11	36 ± 15	20 (95)	20 (87)	13 (62)	9 (39)	N/A	N/A	3.1 ± 5.2	5.6 ± 6.9	N/A	N/A	4.7 (3.2)	5 (3.16)	N/A	N/A	7 (33)	10 (44)	6 (29)	8 (35)	7 (33)	5 (22)	N/A	N/A	N/A	N/A	12.0 ± 2.3	12.5 ± 1.8
McIntyre et al^9^	38	29	39.8 ± 18.1	41.7 ± 20.4	28 (74)	26 (90)	N/A	N/A	N/A	N/A	2.3 (3.85)	2.3 (3.9)	19.7 ± 5.8	19.2 ± 5.2	7.5 ± 3.4	7.3 ± 3.6	N/A	N/A	N/A	N/A	N/A	N/A	N/A	N/A	N/A	N/A	N/A	N/A	N/A	N/A
Robertson^10^ et al	101	99	33.3 (15.8)	32.3 (20.3)	88 (87.1)	85 (85.9)	67 (66.3)	71 (71.7)	22 (21.8)	10 (10.1)	N/A	N/A	N/A	N/A	N/A	N/A	15 (14.9)	7 (7.1)	9 (8.9)	18 (18.2)	17 (16.8)	14 (14.1)	60 (59.4)	60 (60.6)	147.6 (44.64)	158.4 (41.94)	N/A	N/A	14.3 (2)	14.3 (1.96)
Taccone^8^ et al (TRAIN)	397	423	52 (16)	51 (16)	218 (54.9)	226 (53.4)	264 (81.5)	279 (79.9)	57 (24)	50 (20)	N/A	N/A	19 (8)	19 (8)	6.3 (4.46)	6 (4.46)	5 (2)	6 (2)	96 (40)	88 (36)	N/A	N/A	139 (58)	149 (61.3)	165 (54)	162 (58)	140 (5)	140 (6)	11.7 (2.1)	11.7 (2)
Turgeon^11^ et al (HEMOTION)	369	367	48.9 ± 18.8	48.4 ± 19.0	280 (75.88)	255 (69.48)	324 (87.8)	324 (88.3)	65 (17.6)	67 (18.3)	2.7 (1.49)	2.7 (2.23)	N/A	N/A	N/A	N/A	15 (4.1)	25 (6.8)	N/A	N/A	75 (20.3)	71 (19.3)	279 (75.6)	271 (73.84)	165.6 ± 64.8	163.8 ± 68.4	N/A	N/A	13.3 ± 1.8	13.1 ± 1.7
English et al^12^ (SAHaRA)	366	366	59.3 ± 12.5	59.5 ± 12.2	67 (18.3)	67 (18.3)	366 (100)	366 (100)	N/A	N/A	N/A	N/A	N/A	N/A	N/A	N/A	N/A	N/A	N/A	N/A	N/A	N/A	N/A	N/A	N/A	N/A	N/A	N/A	9.4 ± 0.6	9.3 ± 0.6

APACHE II indicates Acute Physiology and Chronic Health Evaluation II score; Lib, liberal strategy; RBC, red blood cells; Res, restrictive strategy; SD: standard deviation.

### Risk of bias and certainty of evidence

**Figure 2.**
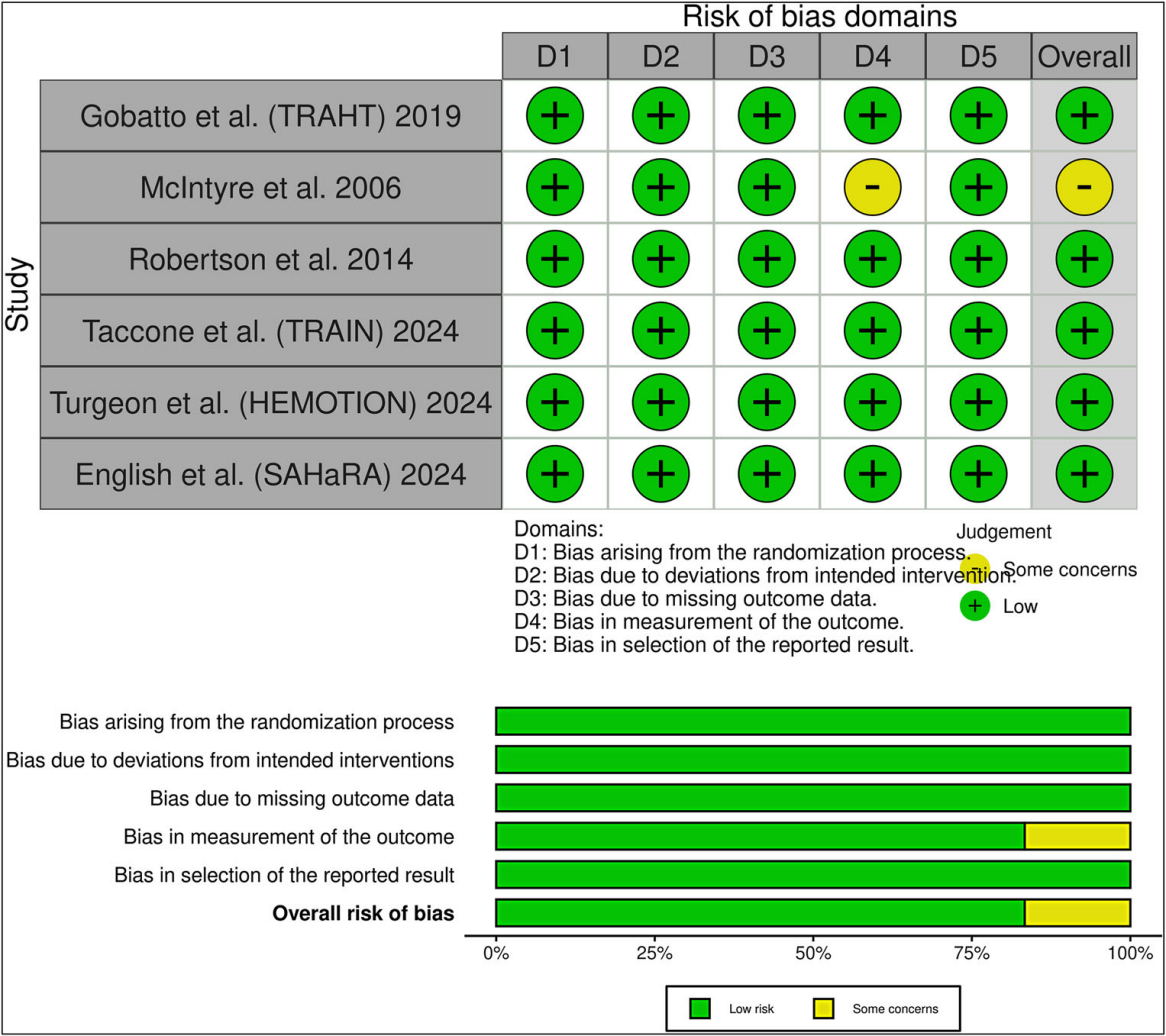
Quality assessment of risk of bias in the included trials. The upper panel presents a schematic representation of risks (low = green, unclear = yellow, and high = red) for specific types of bias in each study in the review. The lower panel presents risks (low = green, unclear = yellow, and high = red) for the subtypes of biases of the combination of studies included in this review.

The risk of bias in each study was assessed using the Cochrane ROB-2 method, with the results summarized in *Figure 2*. Five of the six studies that were evaluated exhibited a low risk of bias across all domains. Nevertheless, the study by McIntyre et al raised some concerns in the fourth domain due to insufficient information on outcome assessors’ blinding. A GRADE evidence profile illustrates the certainty of evidence (*Table 3*).

**Table 3.** GRADE Evidence profile

Certainty assessment							Summary of findings				
Participants (studies)	Risk of bias	Inconsistency	Indirectness	Imprecision	Publication bias	Overall certainty of evidence	Study event rates (%)		Relative effect (95% CI)	Anticipated absolute effects	
							With Restrictive	With Liberal		Risk with Restrictive	Risk difference with Liberal
**Unfavorable neurological outcome**											
2478(5 RCTs)	Not serious	Serious^b^	Serious^d^	Serious^a^	None	⨁◯◯◯ Very Low	725/1242 (58.4%)	658/1236 (53.2%)	**RR 0.90** (0.79 to 1.03)	725/1242 (58.4%)	58 fewer per 1000 (from 123 fewer to 18 more)
**Favorable neurological outcome**											
2478(5 RCTs)	Not serious	Serious^b^	Serious^d^	Serious^a^	None	⨁◯◯◯ Very Low	419/1242 (33.7%)	492/1236 (39.8%)	**RR 1.16** (1.00 to 1.35)	419/1242 (33.7%)	54 more per 1000 (from 0 fewer to 118 more)
**All-cause mortality**											
2566(6 RCTs)	Not serious	Not serious	Not serious	Not serious	None	⨁⨁⨁⨁ High	314/1283 (24.5%)	303/1283 (23.6%)	**RR 0.97** (0.84 to 1.11)	314/1283 (24.5%)	7 fewer per 1000 (from 39 fewer to 27 more)
**Hospital mortality**											
1579(4 RCTs)	Not serious	Not serious	Not serious	Serious^a^	None	⨁⨁⨁◯ Moderate	161/785 (20.5%)	165/794 (20.8%)	**RR 1.02** (0.84 to 1.23)	161/785 (20.5%)	4 more per 1000 (from 33 fewer to 47 more)
**ICU mortality**											
847(3 RCTs)	Not serious	Serious^c^	Not serious	Serious^a^	None	⨁⨁◯◯ Low	66/419 (15.8%)	67/428 (15.7%)	**RR 1.00** (0.73 to 1.37)	66/419 (15.8%)	0 fewer per 1000 (from 43 fewer to 58 more)
**Sepsis or septic shock**											
2532(5 RCTs)	Not serious	Not serious	Not serious	Serious^a^	None	⨁⨁⨁◯ Moderate	115/1278 (9.0%)	81/1254 (6.5%)	**RR 0.73** (0.56 to 0.96)	115/1278 (9.0%)	24 fewer per 1000 (from 40 fewer to 4 fewer)
**Deep venous thrombosis**											
2532(5 RCTs)	Not serious	Not serious	Not serious	Serious^a^	None	⨁⨁⨁◯ Moderate	66/1278 (5.2%)	83/1254 (6.6%)	**RR 1.27** (0.93 to 1.73)	66/1278 (5.2%)	14 more per 1000 (from 4 fewer to 38 more)
**Acute respiratory distress syndrome**											
2532(5 RCTs)	Not serious	Serious^c^	Not serious	Serious^a^	None	⨁⨁◯◯ Low	62/1278 (4.9%)	76/1254 (6.1%)	**RR 1.26** (0.91 to 1.73)	62/1278 (4.9%)	13 more per 1000 (from 4 fewer to 35 more)

CI indicates confidence interval; ICU, intensive care unit; RCT, randomized controlled trial; RR, risk ratio.

^a^ A wide confidence interval that does not exclude the appreciable harm or benefit.

^b^ I^2^ > 50%.

^c^ A wide confidence interval that does not exclude the appreciable harm or benefit, with a low number of events.

^d^ Different scales.

### Primary neurological outcomes

#### Unfavorable neurological outcomes

**Figure 3.**
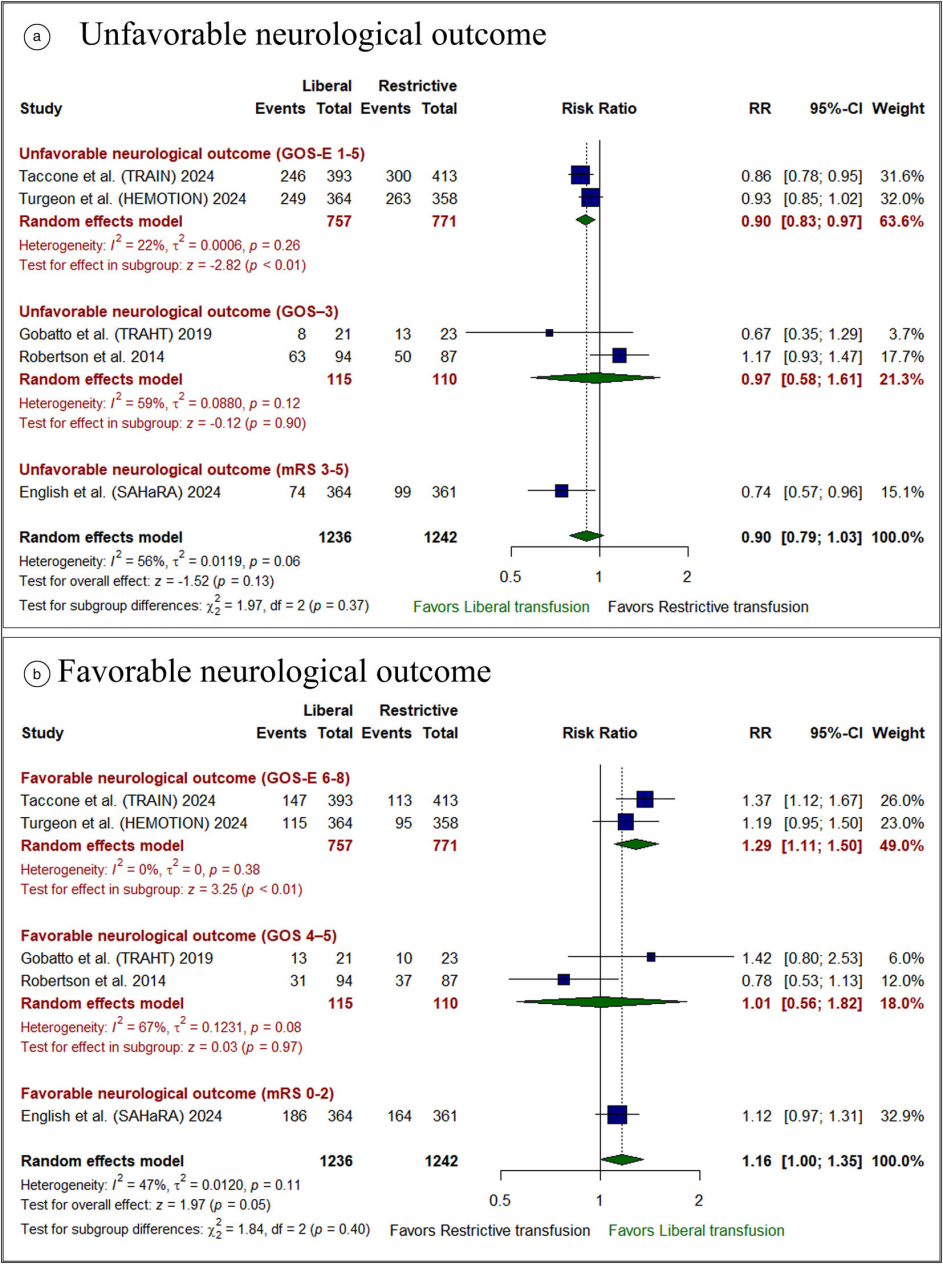
Forest plot of the primary efficacy outcomes: **(a)** unfavorable neurological outcomes and **(b)** favorable neurological outcomes. CI indicates confidence interval; RR, risk ratio.

There was no substantial difference in unfavorable neurological endpoints between the restrictive and liberal transfusion groups (RR: 0.90 [95% CI: 0.79–1.03]; *P* = 0.13). The test of subgroup differences based on the assessment tool was insignificant (*P* = 0.37) (*Figure 3a*). Pooled studies were heterogeneous in the pooled analysis (I^2^ = 56%, *P* = 0.06).

#### Favorable neurological outcomes

There was no marked difference in favorable neurological outcomes between the liberal and restrictive transfusion groups (RR: 1.16 [95% CI: 1.00– 1.35]; *P* = 0.05). The test of subgroup differences based on the assessment tool was insignificant (*P* = 0.40) (*Figure 3b*). Pooled studies were homogeneous in the pooled analysis (I^2^ = 47%, *P* = 0.11).

### Secondary efficacy outcomes

#### Length of stay

There was no notable difference in hospital or ICU length of stay between the liberal and restrictive transfusion groups. The MD for hospital length of stay was −0.66 [95% CI: −2.48–1.16]; *P* = 0.48). Similarly, for ICU length of stay, the MD was 0.15 [95% CI: −0.68–0.99]; *P* = 0.72) *(Supplemental Figure S1)*. The pooled studies were homogenous in hospital and ICU length of stay (I^2^ = 0%, *P* = 0.44, and I^2^ = 43%, *P* = 0.14, respectively).

#### Red-cell units transfused

The liberal transfusion strategy was significantly associated with an increase in the number of red-cell units transfused compared to the restrictive strategy (MD: 2.28, [95% CI: 1.75–2.80], *P* < 0.01) (*Supplemental Figure S2*). The pooled studies were heterogeneous (I^2^ = 94%; *P* < 0.01). Sensitivity analysis failed to resolve the heterogeneity (*Supplemental Figure S3*).

### Safety outcomes

**Figure 4.**
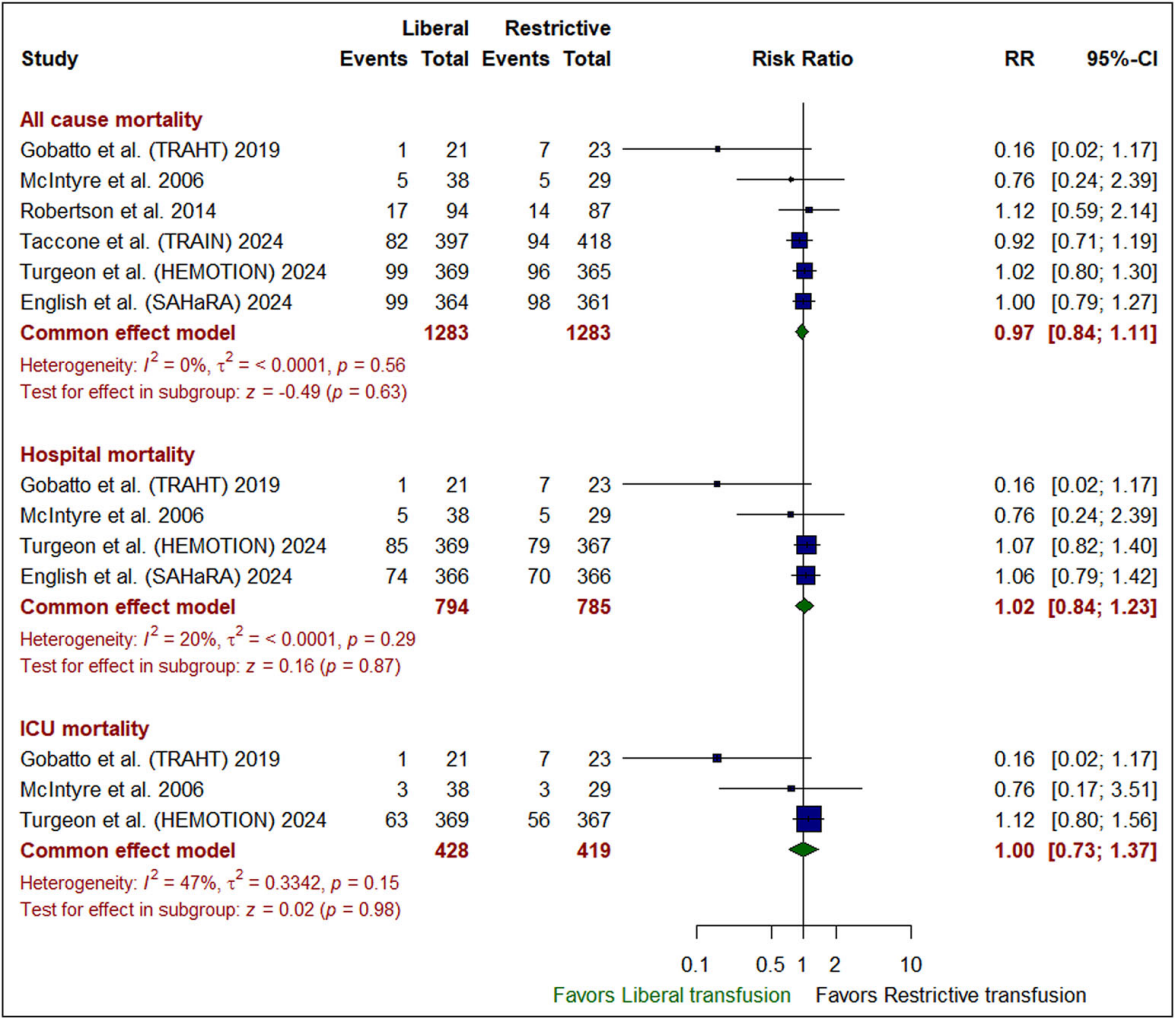
Forest plot for mortality. CI indicates confidence interval; ICU, intensive care unit; RR, risk ratio.

**Figure 5.**
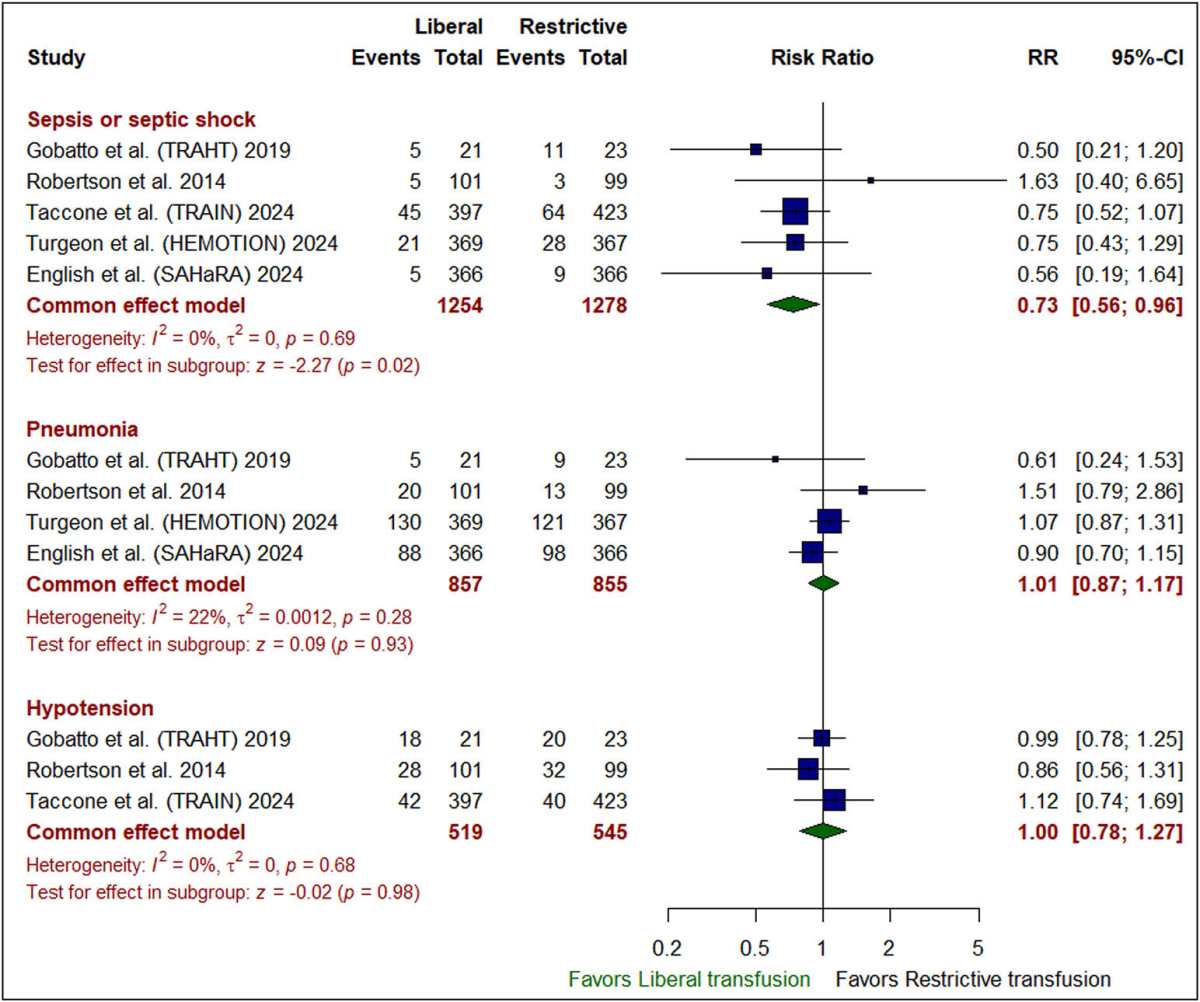
Forest plot for primary safety outcomes. CI indicates confidence interval; RR, risk ratio.

**Figure 6.**
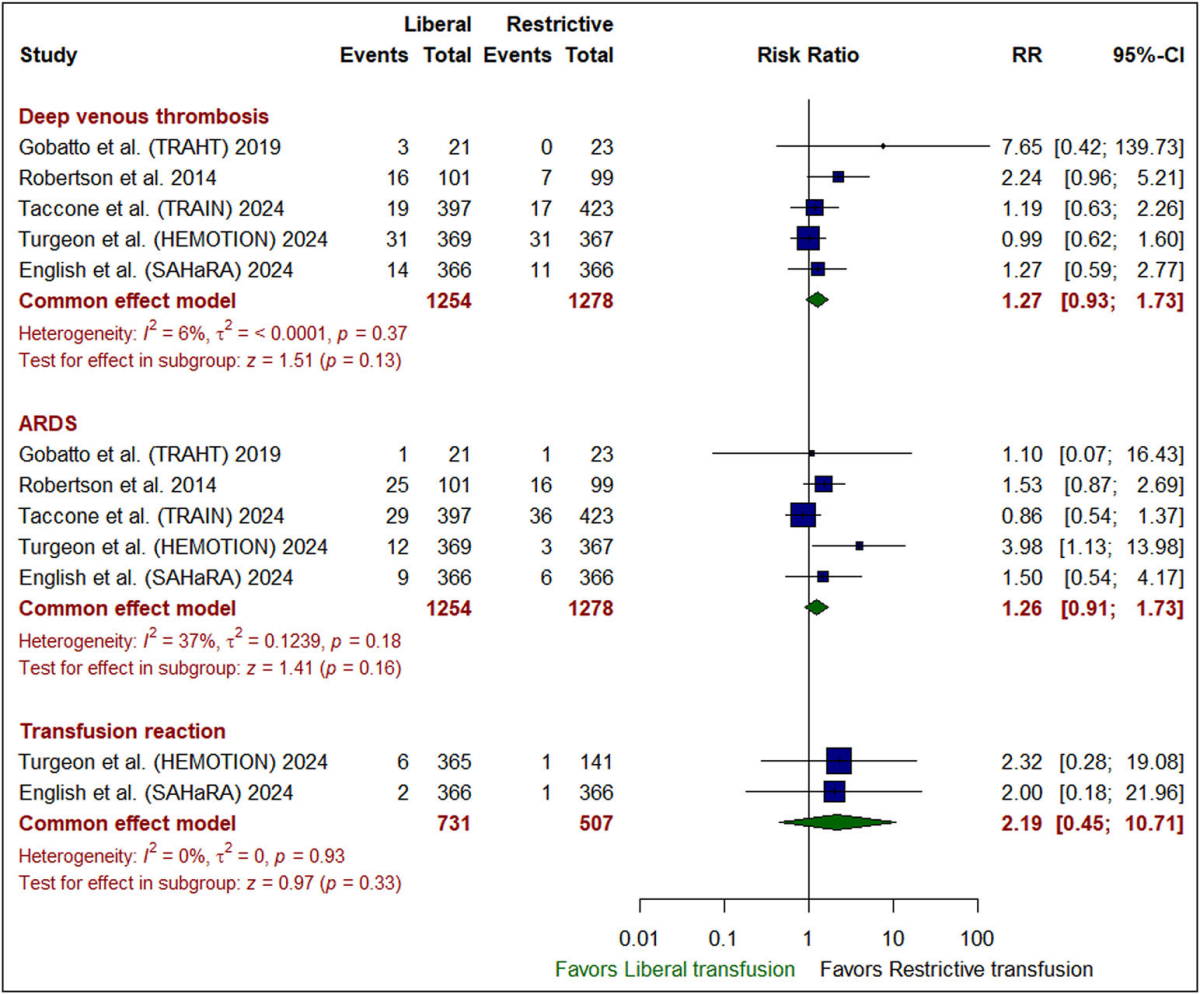
Forest plot for secondary safety outcomes. ARDS indicates acute respiratory distress syndrome, CI, confidence interval; RR, risk ratio.

The liberal transfusion strategy was significantly accompanied with a reduced incidence of sepsis or septic shock compared to the restrictive one (RR: 0.73 [95% CI: 0.56– 0.96]; *P* = 0.02). However, there was no marked variation between the liberal and restrictive transfusion groups in the occurrences of all-cause mortality (RR: 0.97 [95% CI: 0.84–1.11]; *P* = 0.63), hospital mortality (RR: 1.02 [95% CI: 0.84–1.23]; *P* = 0.87), or ICU mortality (RR: 1.00 [95% CI: 0.73–1.37]; *P* = 0.98) **(***Figure 4***)**, pneumonia (RR: 1.01 [95% CI: 0.87–1.17]; *P* = 0.93), hypotension (RR: 1.00 [95% CI: 0.78–1.27]; *P* = 0.98) **(***Figure 5***)**, deep venous thrombosis (RR: 1.27 [95% CI: 0.93–1.73]; *P* = 0.13), acute respiratory distress syndrome (RR: 1.26 [95% CI: 0.91– 1.73]; *P* = 0.16), and transfusion reaction (RR: 2.19 [95% CI: 0.45–10.71]; *P* = 0.33) (*Figure 6*). Pooled studies were homogenous in all endpoints (I^2^ <50%, *P* > 0.1).

## DISCUSSION

Our study observed no remarkable variations in neurological endpoints between liberal and restrictive transfusion strategies. However, the liberal strategy significantly reduced the risk of sepsis and septic shock compared to the restrictive approach. Additionally, the liberal group required transfusion of more red-cell units. There were no variations in the length of hospital or ICU stay among the groups. Finally, there were no substantial variations in all-cause mortality rates or major nonneurological consequences, such as pneumonia, acute respiratory distress syndrome, hypotension, deep venous thrombosis, or transfusion reactions, between the liberal and restrictive transfusion groups. Notwithstanding the lower sepsis/septic shock signal with a liberal strategy, red-cell transfusion carries established risks, acute complications such as transfusion-related acute lung injury and transfusion-associated circulatory overload, and a possible increase in health care–associated infection via transfusion-related immunomodulation. Contemporary international guidance generally favors restrictive thresholds around 7 g/dL for most hospitalized adults (with individualization to physiology and comorbidity). Accordingly, our sepsis finding should be interpreted alongside these recognized harms and the neutral mortality results, reinforcing an individualized, physiology-informed approach.^7^^,^^8^

The results of our meta-analysis contradict the evidence from a prior meta-analysis carried out by Yu et al,^18^ which suggested no difference in the incidence of sepsis and septic shock and a decreased risk of deep venous thrombosis in the restrictive group. However, they agreed that there is no difference regarding neurological outcomes, mortality benefits, length of hospital stay, or risk of major nonneurological complications. The evidence from that previous study was weak and insufficient due to the minimal study populations of the eligible studies and their notable heterogeneity.^18^

Our study, on the other hand, included the most recent and large multicenter RCTs, including the TRAIN trial by Taccone et al,^8^ the HEMOTION trial by Turgeon et al,^11^ and the SAHaRA trial by English et al.^12^ This increased the sample size and the statistical power of our meta-analysis. The largest of these recently published trials is the TRAIN trial, which was conducted in 72 centers across 22 countries with 820 total participants followed up for 180 days to determine the proportion of individuals with unfavorable neurological consequences using the GOS-E and initiating liberal transfusion when hemoglobin is <9 g/dL. The primary outcome results of the TRAIN trial contradicted those of the HEMOTION trial, which involved 736 patients in 34 centers. Both trials used the same scale to assess neurological function (GOS-E) and were conducted over a shorter time frame of 6 months. Differences between the two trials include the hemoglobin transfusion threshold for the liberal group (9 g/dL in TRAIN vs 10 g/dL in HEMOTION), the definition of unfavorable neurological outcome (GOS-E score of 1–5 in TRAIN vs 1–4 in HEMOTION), and the target population (more heterogeneous acute brain injury in TRAIN vs TBI in HEMOTION).^8–11^ The most recently published SAHaRA trial involved 732 participants in 23 centers followed up for 12 months. Cases mainly involved subarachnoid hemorrhage, and they could not detect the superiority of the liberal over the restrictive strategy when using the mRS score for unfavorable neurological outcomes, defined as ≥ 4. However, the results of this trial may be limited due to being open labeled and biased by the risk of vasospasm following subarachnoid hemorrhage.^12^ While our meta-analysis did not find statistically significant differences in neurological outcomes overall, it is worth noting that the TRAIN trial reported a substantial reduction in the rate of unfavorable neurological outcomes, an absolute difference of approximately 10% with an adjusted relative risk of 0.86 (95% CI: 0.79–0.94; *P* = 0.002), in favor of the liberal transfusion strategy (hemoglobin < 9 g/dL).^8^ Although the SAHaRA trial in subarachnoid hemorrhage did not show a statistically significant benefit, it did indicate a consistent trend toward improved outcomes with the liberal approach (RR 0.88; 95% CI: 0.72–1.09; *P* = 0.22).^12^ These signals suggest potential clinical benefits in selected populations and underscore the urgent need for further targeted, high-quality trials to determine whether liberal transfusion thresholds can yield meaningful benefit in specific acute brain injury groups.

The lack of difference in mortality between both groups may be attributed to receiving the same overall medical interventions. Additionally, the absence of difference in most significant complications can be due to similar care intensity provided to both groups, resulting in a similar length of stay in the ICU or hospital. The better neurological outcomes in survivors in the liberal group may result from a lower incidence of cerebral infarctions in this group.^8^ Our brains are susceptible to hypoxia and typically have compensatory mechanisms to maintain adequate oxygenation to cerebral tissue, which depends on cerebral blood flow and arterial oxygen content.^19^ The brain can compensate by dilating blood vessels to increase cerebral blood flow because the oxygen uptake by the brain is usually high, so the effect of increasing oxygen uptake is limited. This regulation of cerebral blood flow is mainly caused by the carbon dioxide effect, which is responsible for cerebral blood vessel reserve.

Patients with acute brain injuries have impaired regulation mechanisms and insufficient cerebrovascular reserve, and brain hypoxia may occur even with elevated hemoglobin levels.^20^^,^^21^ A previous prospective study suggests a relation between defective cerebrovascular reactivity and the risk of cerebral ischemic insults.^22^ In cases of severe anemia with hemoglobin levels <5 g/dL, this mechanism cannot further deal with brain hypoxia. Besides direct blood loss, many factors may cause anemia in critical patients. These include dilutional anemia from aggressive fluid resuscitation and reduced RBC production due to impaired metabolism of folic acid, vitamin B12, iron, or decreased erythropoietin. Elevated levels of inflammatory cytokines cause these changes. The systemic inflammatory reaction in severe cases can further shorten the lifespan of RBCs by oxidative stress-induced apoptosis of RBCs.^23^^,^^24^

Taken together, these findings suggest that transfusion thresholds may need to be tailored by injury type. While liberal strategies showed signals of benefit in heterogeneous acute brain injury (TRAIN)^8^ and a trend in subarachnoid hemorrhage (SAHaRA),^12^ no such advantage was evident in TBI (HEMOTION).^11^ This variability underscores that a uniform restrictive threshold may not be optimal across all etiologies and supports an individualized, physiology-guided approach.^2^^,^^6^^,^^20^

A previous retrospective study suggested that baseline hypoxia can better predict oxygenation improvement with transfusion than the hemoglobin baseline. In 41% of patients, transfusion improved brain tissue oxygenation.^25^ Some studies have indicated that using blood hemoglobin levels as the only guide for blood transfusion is not the most appropriate approach and have suggested using other indicators and monitoring tools like noninvasive cerebral oxygenation measurements or positron emission tomography.^26^^,^^27^

### Strengths and limitations

Our meta-analysis included six high-quality and recent RCTs with 2599 participants in 146 centers worldwide. However, several limitations should be acknowledged. First, substantial heterogeneity was observed in the GOS and red-cell units transfused, and sensitivity analyses were unable to fully account for these differences. This heterogeneity likely reflects variations in study populations, injury etiologies, transfusion triggers, and cointerventions across trials. Second, follow-up durations varied between studies, which may influence the comparability of long-term outcomes. Third, different scales and definitions were used to measure neurological and clinical outcomes, potentially affecting pooled estimates. Fourth, the open-label design of all included trials, necessitated by the nature of the intervention, introduces the possibility of performance and detection bias, particularly for subjective outcomes, and may have influenced cointerventions or clinical decision-making. Also, definitions of restrictive and liberal RBC transfusion thresholds varied between studies, which could have contributed to both clinical and statistical heterogeneity. Fifth, we did not assess publication or reporting bias because only six trials were available for inclusion. As recommended in the Cochrane Handbook, conventional tools such as funnel plots and Egger’s test are underpowered with fewer than 10 studies, and therefore results would be unreliable. While our comprehensive search strategy likely minimized the risk of missed studies, the possibility of unpublished negative trials cannot be excluded.

Lastly, although subgroup analyses by etiology (e.g., TBI, subarachnoid hemorrhage, intracerebral hemorrhage) and transfusion threshold could offer further clinical insights, we did not perform such analyses for methodological reasons. The limited number of RCTs for each specific etiology, coupled with mixed populations in several studies and absence of outcome reporting stratified by diagnosis, would have resulted in underpowered comparisons with wide confidence intervals, increasing the risk of spurious findings. To preserve statistical validity and adhere to our prespecified protocol, we synthesized results at the overall acute brain injury level. This approach may limit the applicability of our findings to individual subgroups, underscoring the need for future adequately powered RCTs targeting specific etiologies and transfusion strategies.

### Implications for future research

Upcoming research should prioritize establishing more multicenter, double-blinded RCTs to validate this meta-analysis’s findings further. These studies should aim to standardize the definitions of restrictive and liberal transfusion thresholds and use consistent outcome measures, such as the GOS-E and mRS, to facilitate comparison across studies. Exploring the underlying mechanisms of how liberal transfusion strategies improve neurological outcomes could provide valuable insights. Investigating the role of noninvasive cerebral oxygenation monitoring and other advanced imaging techniques in guiding transfusion decisions may also enhance patient care. Finally, research should consider the long-term effects of transfusion strategies on both neurological and nonneurological outcomes to provide thorough insight into their benefits and risks.

## Conclusion

The liberal strategy of blood transfusion for patients suffering from acute brain injury and anemia did not impact neurological outcomes. However, it reduced the incidence of sepsis or septic shock, but this came with an increase in the number of RBCs transfused without affecting the overall mortality and thrombotic events. While sepsis reduction is clinically relevant, the absence of improvement in neurological outcomes or survival tempers its impact, emphasizing that transfusion triggers should remain individualized and physiology-informed in line with current guidance. More multicenter, double-blinded trials are required to confirm these findings.

## Supplementary Material

Supplemental Material
