# Supplementary material for: Liberal versus restrictive transfusion strategies in patients with acute brain injury: a systematic review and meta-analysis of randomized controlled trials
Source: Proc (Bayl Univ Med Cent). 2025 Dec 10;39(1):139–51. doi: 10.1080/08998280.2025.2586988 (PMC12778877; doi:10.1080/08998280.2025.2586988)
Supplement: Supplemental Material [file UBMC_A_2586988_SM3181.docx]

**Supplemental Material**

**Liberal versus restrictive transfusion strategies in patients with acute brain injury: a systematic review and meta-analysis of randomized controlled trials**

Mazen Alayidh, AlMothana M. Manasrah, Ahmed A. Maiz, Mohamed Rifai, Ahmed A. Ibrahim, Shaden Alayidh, Sara A. Al Asheikh, Ali Alaklah, Mohamed Saad Rakab, Mustafa Turkmani, and Mohamed Abuelazm

***Baylor University Medical Center Proceedings* 2026;39(1)**

Table S1: Search strategy.

| **Database** | **Search Terms** | **Result** |
| --- | --- | --- |
| **PubMed** | (“brain injury” OR “traumatic brain injury” OR “TBI” OR “acute brain injury” OR “head trauma”) AND (“Blood Transfusion” OR “Transfusion” OR “Red Blood Cell Transfusion” OR “transfusion threshold”) AND (“restrictive” OR “restrictive transfusion” OR “restrictive blood transfusion” OR “restrictive strategy” OR “hemoglobin ≤7”) AND (“liberal” OR “liberal transfusion” OR “liberal blood transfusion” OR “hemoglobin ≤10”) | 32 |
| **CENTRAL** | (“brain injury” OR “traumatic brain injury” OR “TBI” OR “acute brain injury” OR “head trauma”) AND (“Blood Transfusion” OR “Transfusion” OR “Red Blood Cell Transfusion” OR “transfusion threshold”) AND (“restrictive” OR “restrictive transfusion” OR “restrictive blood transfusion” OR “restrictive strategy” OR “hemoglobin ≤7”) AND (“liberal” OR “liberal transfusion” OR “liberal blood transfusion” OR “hemoglobin ≤10”) | 18 |
| **Scopus** | (“brain injury” OR “traumatic brain injury” OR “TBI” OR “acute brain injury” OR “head trauma”) AND (“Blood Transfusion” OR “Transfusion” OR “Red Blood Cell Transfusion” OR “transfusion threshold”) AND (“restrictive” OR “restrictive transfusion” OR “restrictive blood transfusion” OR “restrictive strategy” OR “hemoglobin ≤7”) AND (“liberal” OR “liberal transfusion” OR “liberal blood transfusion” OR “hemoglobin ≤10”) | 38 |
| **Web of Science** | (“brain injury” OR “traumatic brain injury” OR “TBI” OR “acute brain injury” OR “head trauma”) AND (“Blood Transfusion” OR “Transfusion” OR “Red Blood Cell Transfusion” OR “transfusion threshold”) AND (“restrictive” OR “restrictive transfusion” OR “restrictive blood transfusion” OR “restrictive strategy” OR “hemoglobin ≤7”) AND (“liberal” OR “liberal transfusion” OR “liberal blood transfusion” OR “hemoglobin ≤10”) | 44 |
| **EMBASE** | (“brain injury” OR “traumatic brain injury” OR “TBI” OR “acute brain injury” OR “head trauma”) AND (“Blood Transfusion” OR “Transfusion” OR “Red Blood Cell Transfusion” OR “transfusion threshold”) AND (“restrictive” OR “restrictive transfusion” OR “restrictive blood transfusion” OR “restrictive strategy” OR “hemoglobin ≤7”) AND (“liberal” OR “liberal transfusion” OR “liberal blood transfusion” OR “hemoglobin ≤10”) | 60 |


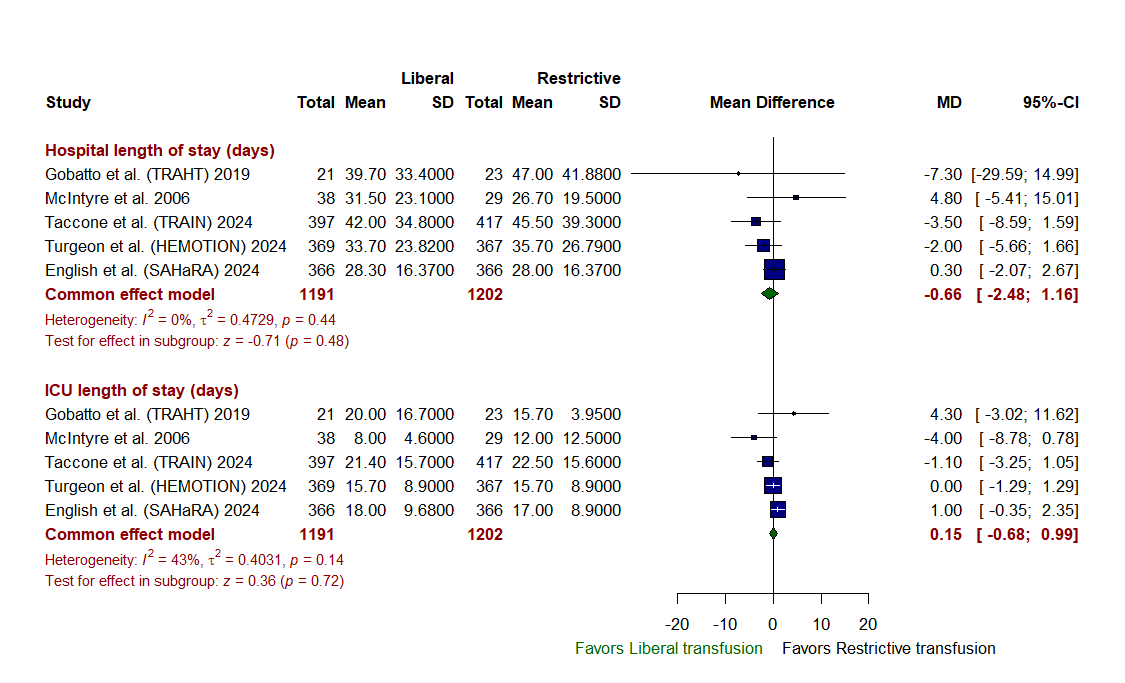
**Figure S1: Forest plot of hospital and ICU Length of Stay.**


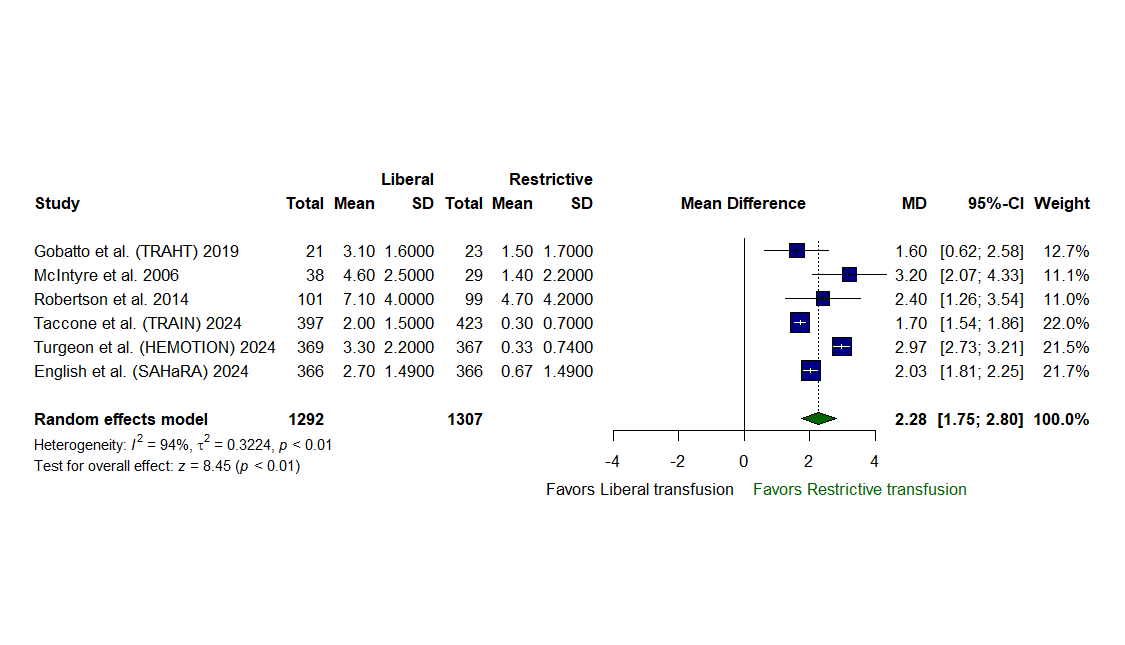


Figure S2: Forest plot of red-cell units transfused.


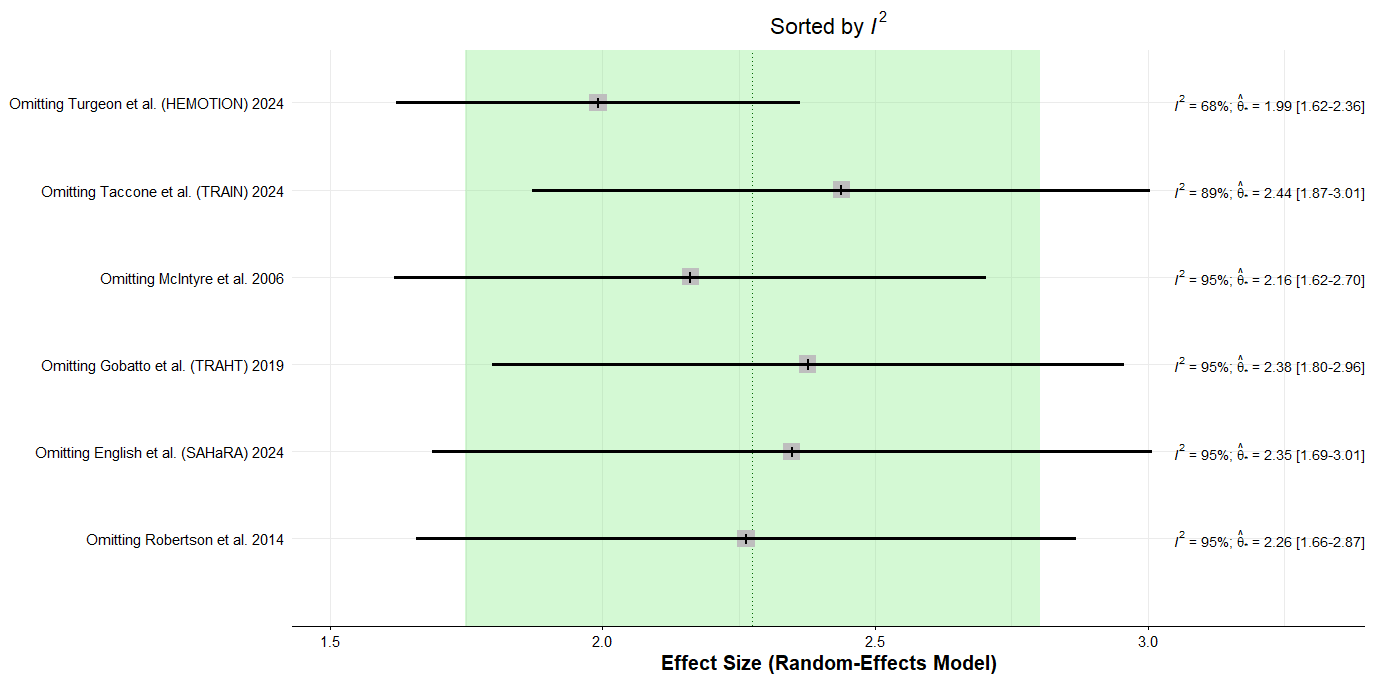


Figure S3: Sensitivity analysis of red-cell units transfused.

**PRISMA 2020 Checklist**

| **Section/Topic** | **#** | **Checklist item** | **Location in manuscript** | **Status / Action** |
| --- | --- | --- | --- | --- |
| TITLE | 1 | Identify the report as a systematic review/meta-analysis. | Title page (ensure the phrase “systematic review and meta‑analysis” is included). | Reported. |
| ABSTRACT | 2 | Provide a structured abstract (background, objectives, data sources, eligibility, participants/interventions, methods, results, limitations, conclusions, registration). | Abstract (structured) — add PROSPERO ID. | Reported. |
| INTRODUCTION | 3 | Rationale: describe why the review is needed. | Sec 1.0 Introduction, | Reported. |
| INTRODUCTION | 4 | Objectives: state explicit objectives/questions. | Sec 1.0 Introduction, last ¶ ("To explore these conflicting results…"). | Reported. |
| METHODS | 5 | Eligibility criteria (PICO, study designs, settings, report characteristics). | Sec 2.3 Eligibility Criteria. | Reported. |
| METHODS | 6 | Information sources (databases, registries, other sources; last search date). | Sec 2.2 Evidence Sources & Retrieval Strategy (search to 10 Feb 2025). | Reported. |
| METHODS | 7 | Search strategy: present full strategies for all databases. | Table S1 (search strings). | Reported. |
| METHODS | 8 | Selection process: how studies were selected; reviewers, automation tools; independence. | Sec 2.4 Study Selection (Covidence; independent reviewers; consensus with third author). | Reported. |
| METHODS | 9 | Data collection process: extraction methods, piloting, reviewers, adjudication. | Sec 2.5 Data Extraction (pilot; independent extractors; adjudication). | Reported. |
| METHODS | 10a | Data items — prespecified outcomes and definitions. | Sec 2.3 Outcomes (primary and secondary). | Reported. |
| METHODS | 10b | Data items — other variables (participant, intervention, study characteristics). | Sec 2.5 (baseline and study characteristics list). | Reported. |
| METHODS | 11 | Study risk of bias assessment (tools, domains, reviewers, process). | Sec 2.6 Risk of Bias and Certainty (RoB2; independent reviewers). | Reported. |
| METHODS | 12 | Effect measures for each outcome (e.g., RR, MD) and direction of effect. | Sec 2.7 Statistical Analysis. | Partially reported. |
| METHODS | 13a | Synthesis methods — how studies were grouped/decided eligible for each synthesis. | Sec 2.7; Sec 3.4 (by outcome; subgroups by scale). | Reported. |
| METHODS | 13b | Data preparation (e.g., handling of missing data, conversions). | Sec 2.7 (continuity correction 0.5). | Reported. |
| METHODS | 13c | Methods to tabulate or visually display results. | Sec 2.7; Figures 3–6; Figures S1–S3. | Reported. |
| METHODS | 13d | Statistical synthesis methods (model choice, heterogeneity statistics, software, τ² estimator). | Sec 2.7 (random-/fixed‑effect rules; I²/χ²; R v4.3; meta/metafor/dmetar). | Reported. |
| METHODS | 13e | Methods to explore heterogeneity (subgroup/meta‑regression) and rationale. | Sec 2.7 (planned subgroup by scale); Sec 3.4 (Q_between / subgroup test). | Reported. |
| METHODS | 13f | Sensitivity analyses (what was planned; how conducted). | Sec 2.7; Sec 3.5.1.2; Figure S3. | Reported. |
| METHODS | 14 | Reporting bias assessment (publication/small‑study bias). | — | Reported — stated it was not assessed due to <10 studies and added a note in Discussionesults. |
| METHODS | 15 | Certainty (GRADE) methods. | Sec 2.6; Table 3 (GRADE). | Reported. |
| RESULTS | 16a | Study selection: numbers at each stage; flow diagram. | Sec 3.1; Figure 1 (PRISMA flow). | Reported. |
| RESULTS | 16b | Excluded studies with reasons. | Briefly mentioned in the section 3.1. Search Results and Study Selection. | Partially reported. |
| RESULTS | 17 | Study characteristics of included studies. | Sec 3.2; Tables 1–2. | Reported. |
| RESULTS | 18 | Risk of bias in studies. | Sec 3.3; Figure 2 (RoB2 summary). | Reported. |
| RESULTS | 19 | Results of individual studies (summary data and effect estimates). | Figures 3–6 (forest plots include per‑study estimates). | Reported. |
| RESULTS | 20a | Results of syntheses — summary of contributing studies’ characteristics and ROB. | Sec 3.2–3.3. | Reported. |
| RESULTS | 20b | Statistical syntheses — pooled effects, CIs, heterogeneity, direction, prediction intervals (if used). | Sec 3.4–3.5; Figures 3–6; S1–S2. | Reported. |
| RESULTS | 20c | Heterogeneity exploration results. | Sec 3.4 (subgroup tests). | Reported. |
| RESULTS | 20d | Sensitivity analyses results. | Sec 3.5.1.2; Figure S3. | Reported. |
| RESULTS | 20e | Reporting bias assessment results. | — | Reported. |
| DISCUSSION | 23a | Interpretation in context of other evidence. | Sec 4.0 Discussion, first paragraphs. | Reported. |
| DISCUSSION | 23b | Limitations of included evidence. | Strengths and Limitations (within Discussion). | Reported. |
| DISCUSSION | 23c | Limitations of review processes. | Strengths and Limitations (language restriction; heterogeneity; open‑label). | Reported. |
| DISCUSSION | 23d | Implications for practice/policy/research. | Implications for Future Research; Conclusion. | Reported. |
| OTHER INFORMATION | 24a | Registration. | Sec 2.1 (PROSPERO CRD42025630392). | Reported. |
| OTHER INFORMATION | 24b | Protocol access. | Sec 2.1. | Reported. |
| OTHER INFORMATION | 24c | Amendments to protocol. | Sec 2.1. | Reported. |
| OTHER INFORMATION | 25 | Support/funding and roles of funders. | Declarations/Support section. | Reported. |
| OTHER INFORMATION | 26 | Competing interests. | Competing Interests statement. | Reported. |
| OTHER INFORMATION | 27 | Availability of data, code, and other materials. | Data/code/materials availability statement. | Reported. |
